# Peripartum aortic dissection: a rare case of Stanford type B dissection triggered by severe labor pain

**DOI:** 10.1186/s43044-025-00704-5

**Published:** 2025-11-16

**Authors:** Yun Lu, Zhu Wang, Hu Zhang, Zhongxin Zhou, Jun Wei, Hao Zhang

**Affiliations:** 1 XuZhou Clinical School of Xuzhou Medical University, Xuzhou, China; 2 Affiliated Hospital of Xuzhou Medical College, Xuzhou, China

**Keywords:** Aortic dissection, Stanford type B, Peripartum period, Labor pain, Pregnancy, Connective tissue disease, Multidisciplinary team screening

## Abstract

**Background:**

Peripartum aortic dissection (AD) is an uncommon yet significant complication during pregnancy and delivery, characterized by a tear in the aortic intima that can lead to severe maternal morbidity and mortality. This case report describes a rare instance of Stanford Type B aortic dissection occurring in a pregnant woman, emphasizing that acute, severe labor pain can obscure the diagnosis of AD, often leading to misattribution of symptoms to more common obstetric complications.

**Case presentation:**

This report highlights the case of a 36-year-old primigravida who presented with acute chest pain during labor, initially misdiagnosed until imaging revealed the dissection post-delivery. The critical interplay between labor-induced hemodynamic stress and pre-existing vascular weaknesses, such as those found in connective tissue disorders, raises concerns about the cardiovascular risks faced by pregnant individuals. The importance of a multidisciplinary approach, involving obstetricians, cardiologists, and anesthesiologists, is underscored, as effective management strategies are essential to ensure maternal and fetal safety.

**Conclusion:**

This case underscores the necessity for healthcare providers to maintain a high index of suspicion for atypical thoracic pain in pregnant patients and advocates for enhanced screening protocols for aortic disease. The findings advocate for regular cardiovascular assessments in women of childbearing age with known risk factors to improve early diagnosis and intervention, thereby potentially reducing the associated morbidity and mortality from this rare but critical condition. Further research is needed to develop evidence-based guidelines for managing AD in the peripartum setting, aiming to refine clinical protocols and optimize patient outcomes.

**Supplementary Information:**

The online version contains supplementary material available at 10.1186/s43044-025-00704-5.

## Introduction

Peripartum aortic dissection (AD) presents a unique and critical challenge in obstetric care, characterized by a tear in the intimal layer of the aorta that typically occurs during or immediately after delivery. This condition is particularly alarming, as it is associated with significant maternal morbidity and mortality. The estimated incidence of aortic dissection during pregnancy is approximately 0.4% of all cases, with a notably high maternal mortality rate due to diagnostic challenges and the potential for rapid clinical deterioration [1, 2]. Given that aortic dissection is predominantly observed in older adults, its occurrence in pregnant women is exceedingly rare and often overlooked, necessitating heightened awareness among healthcare providers [3].

The clinical presentation of peripartum aortic dissection often includes acute, severe chest pain that may mimic other obstetric complications such as pulmonary embolism or placental abruption, resulting in diagnostic delays [1, 2]. Patients may also exhibit signs of hemodynamic instability, with differential blood pressures in the limbs and respiratory distress, complicating the identification of the underlying etiology. The non-specific nature of symptoms in this context underscores the need for a high index of suspicion, particularly when faced with atypical pain during labor [4, 5].For early detection of acute aortic dissection during labor, the following clinical tools and indicators are particularly valuable: (1) Blood pressure monitoring: A >20 mmHg difference in bilateral arm blood pressure measurements serves as a crucial warning sign; (2) Pain characterization: ‘Tearing’ or ‘migrating’ chest/back pain distinguishable from routine labor contractions; (3) Bedside echocardiography: Enables rapid assessment of aortic root and ascending aortic diameter, pericardial effusion, and aortic valve regurgitation; (4) ECG monitoring: Non-specific ST-T changes may indicate myocardial ischemia secondary to aortic dissection.

This case report describes a rare instance of Stanford Type B aortic dissection occurring in a pregnant woman, emphasizing the critical role of severe labor pain as a potential trigger for this life-threatening condition. The presentation of acute chest pain in the context of delivery serves as a reminder of the vulnerabilities faced by patients with pre-existing aortic wall abnormalities, necessitating close monitoring and proactive cardiovascular screening in high-risk populations prior to conception [1]. Furthermore, this case highlights the importance of multidisciplinary collaboration among obstetricians, cardiologists, and surgeons to navigate the complexities of managing such emergencies effectively [2, 3].

## Case presentation

### Patient information

We present a case of a 36-year-old primigravida with no significant past medical history, who was admitted at 39 weeks of gestation due to the onset of active labor. The patient had a history of gestational hypertension, with peak systolic blood pressure exceeding 190 mmHg. Notably, she did not exhibit any Marfan-like features such as arachnodactyly or an arm span greater than height. There was no family history of cardiovascular disease or sudden death.Following diagnosis of gestational hypertension, the patient received labetalol 100 mg orally twice daily for blood pressure control, maintaining pre-delivery blood pressure within 140–150/90–100 mmHg range. Oral medication was suspended upon hospital admission for labor initiation, with transition to intravenous blood pressure monitoring.

### Clinical findings

Approximately six hours into labor, during a period of intense contractions, the patient experienced a sudden onset of “tearing” chest pain that was distinct from the labor pain, radiating to her back, accompanied by acute dyspnea and diaphoresis. Blood pressure readings revealed a significant disparity, with the right arm measuring 190/100 mmHg, while the left arm exhibited a weaker pulse and a blood pressure of 140/85 mmHg.

### Diagnostic assessment

Physical examination revealed a newly developed diastolic murmur consistent with aortic regurgitation at the right upper sternal border. An electrocardiogram demonstrated nonspecific ST-T wave changes. Given the high clinical suspicion of aortic dissection (AD), a bedside focused transthoracic echocardiogram was promptly performed, indicating an approximately 35 mm diameter of the descending aorta, with no abnormalities noted in the ascending aorta. An emergency computed tomography angiography (CTA) subsequently confirmed the diagnosis of Stanford type B aortic dissection, originating distal to the left subclavian artery, just above the sinotubular junction and extending into the descending aorta.

### Therapeutic intervention

**Fig. 1:**
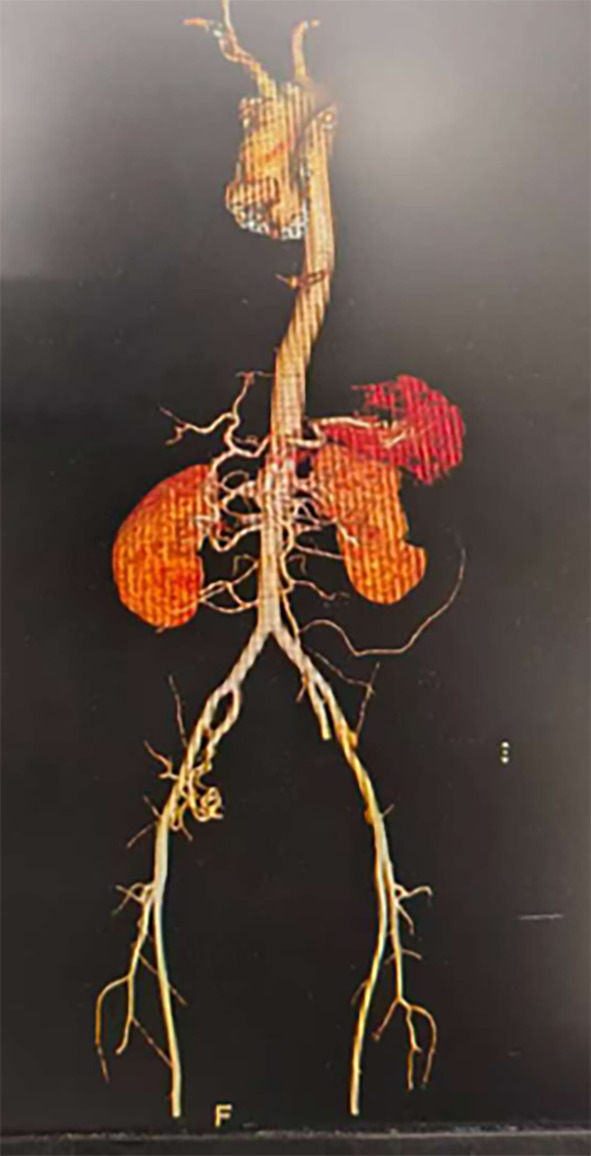
Preoperative computed tomography angiography (CTA) image of the patient.

**Fig. 2:**
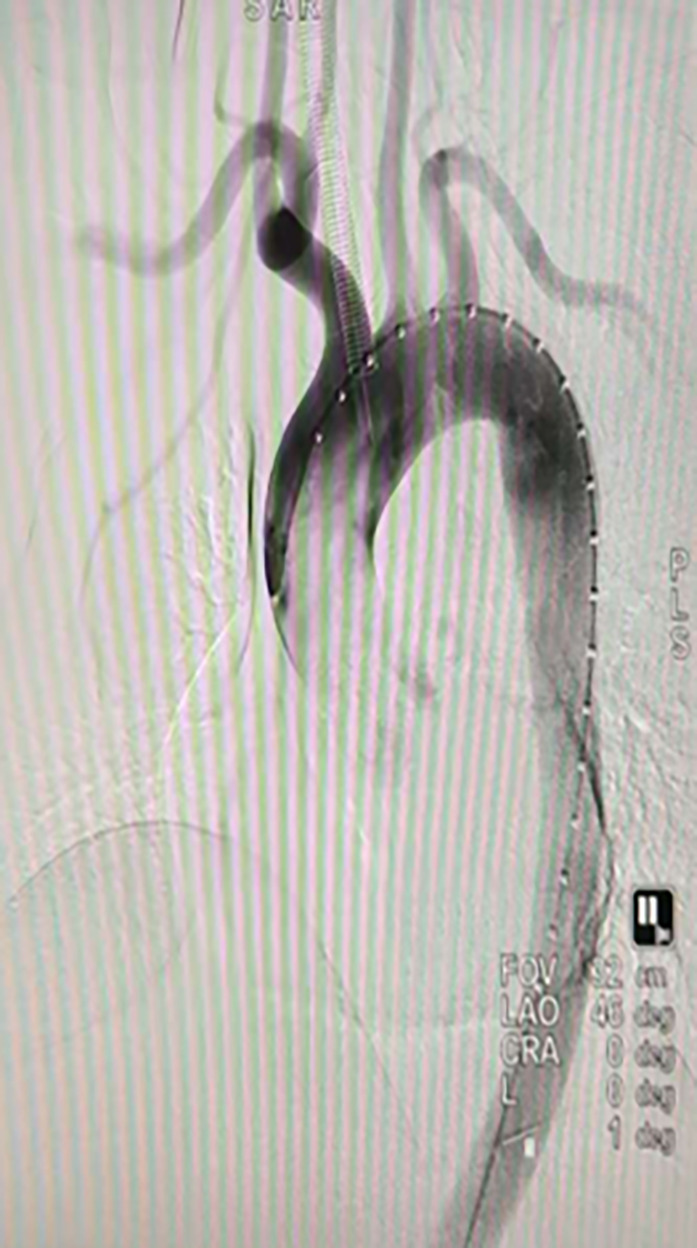
Intraoperative angiography for localization of the dissection tear.

The hospital’s emergency protocol was activated, and a multidisciplinary team (MDT) was assembled, including obstetricians, cardiothoracic surgeons, anesthesiologists, and intensivists. An emergency cesarean section was performed, resulting in the successful delivery of a healthy infant. Following conservative management for two weeks, the patient underwent an elective endovascular aortic stent-grafting procedure. The CTA indicated that the aortic entry tear was located on the concave side of the aorta beyond the left subclavian artery. Given the patient’s type III aortic arch, a tailored approach was taken to ensure the release angle of the stent and maintain the arch’s stability through an external fenestration technique(Figs. 1 and 2). The external fenestration technique represents a modified approach specifically designed for type III aortic arch anatomy. Using Oscor curved guidewires for guidance, we established an 8 mm artificial fenestration 1.5 cm distal to the left subclavian artery origin via an extravascular approach prior to stent deployment. This technique ensured complete coverage of the primary entry tear while maintaining left subclavian artery patency post-stent placement. This approach is particularly suitable for anatomical variations with aortic arch angles exceeding 60°.

**Fig. 3:**
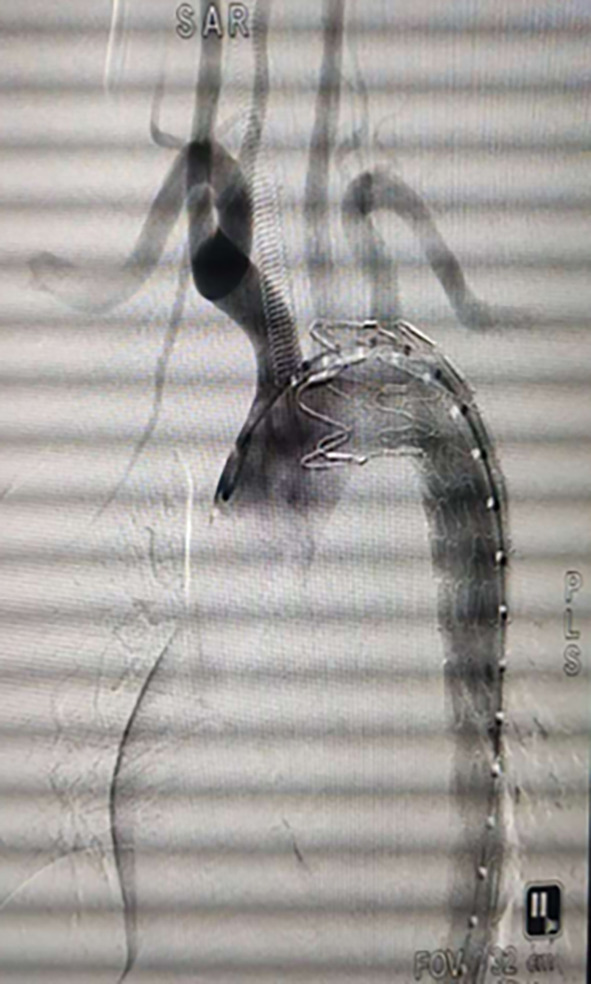
Postoperative angiography following aortic stent-graft implantation.

Post-operative imaging confirmed successful closure of the aortic tear, with normal visualization of the aortic arch branches(Fig. 3). The patient experienced mild postoperative fever, attributed to hematoma absorption, and was treated with antibiotics, antihypertensives, and rate-controlling medications.

## Follow-up and outcomes

After extubation, the patient was transferred back to the ward.The newborn underwent immediate evaluation by our pediatric team, with Apgar scores of 8 and 9 at 1 and 5 min, respectively. No respiratory distress was observed given term gestation and appropriate birth weight (3250 g). Routine cranial ultrasound excluded intracranial hemorrhage, while arterial oxygen saturation was maintained at 95–98%. Standard newborn hearing screening and critical congenital heart disease screening were completed during hospitalization, both returning normal results. Both mother and infant were discharged in good condition two weeks later, with recommendations for genetic testing upon discharge.

At the three-month follow-up, the patient was well-managed on beta-blockers and calcium.The patient described being initially overwhelmed by fear upon hearing the term aortic dissection but expressed profound relief following the successful multidisciplinary intervention, stating that knowing the real cause and having it fixed brought immense peace to our family.

**Table 1:** Clinical timeline of diagnosis and management

Date/Time	Timeline Milestone	Event Description	Clinical Significance
June 17, 2025(Admission)	Hospital Admission	Patient admitted at 39 weeks gestation in active labor.	Initiation of obstetric care.
~ 6 h after labor onset	Symptom Onset	Sudden onset of “tearing” chest pain radiating to back, with dyspnea and diaphoresis.	Key diagnostic clue suggesting aortic dissection, distinct from labor pain.
~ 30 min after pain	Initial Assessment	Physical exam revealed BP disparity (190/100 mmHg R arm vs. 140/85 mmHg L arm) and new diastolic murmur.	Findings highly suspicious for acute aortic syndrome, prompting urgent imaging.
~ 1 h after pain	Bedside Echo	Focused TTE showed descending aorta dilation (~ 35 mm).	Initial imaging corroborated clinical suspicion, guiding next steps.
~ 2 h after pain	Definitive Diagnosis	Emergency CTA confirmed Stanford Type B aortic dissection.	Confirmed diagnosis, enabling immediate multidisciplinary team (MDT) activation.
~ 3 h after pain	MDT Intervention	Emergency cesarean section performed; healthy infant delivered.	Priority: fetal delivery to enable definitive maternal treatment and stabilize hemodynamics.
Post-op Day 1	Postpartum Stabilization	Patient transferred to ICU for close monitoring and BP control.	Critical period for maternal stabilization prior to definitive repair.
Post-op Day 14	Definitive Treatment	Elective endovascular stent-grafting performed via external fenestration technique.	Definitive repair delayed to mitigate surgical risks postpartum.
Post-op Day 16	Recovery	Post-stent imaging confirmed successful closure of entry tear; patient afebrile.	Procedure success confirmed; patient prepared for discharge.
Post-op Day 30	Discharge	Mother and infant discharged in good condition on beta-blockers and antihypertensives.	Successful outcome of multidisciplinary peripartum management.
3-month follow-up	Outpatient Visit	Patient reported no complications; medication well-tolerated.	Favorable medium-term outcome under medical management.

## Discussion

The clinical value of reporting this case lies not only in its rarity but also in its potential to inform medical practice regarding the management of aortic dissection in pregnant patients. The importance of recognizing atypical pain as a possible sign of aortic dissection could lead to earlier diagnosis and intervention, thereby improving maternal and fetal outcomes. This case serves as a crucial reminder of the cardiovascular risks associated with labor and the necessity of implementing improved screening and management protocols for pregnant women at risk of aortic dissection. It is essential for healthcare systems to adopt strategies that involve regular cardiovascular assessments for women of childbearing age, particularly those with known risk factors, to mitigate the risks associated with this serious condition.

Acute aortic dissection (AD) during the peripartum period presents unique diagnostic challenges, particularly due to the overlap of symptoms with common obstetric conditions. The case at hand demonstrates how severe labor pain can mimic or obscure the presentation of AD, potentially leading to misdiagnosis. For instance, a 36-year-old primigravida developed acute proximal type A AD during labor, where initial symptoms were attributed to typical obstetric complications until imaging revealed the dissection post-delivery [1]. Similarly, another study highlighted a 36-year-old parturient whose AD was initially missed, resulting in severe postoperative complications [2]. These cases underscore the critical need for heightened clinical suspicion in pregnant patients presenting with atypical thoracic pain, particularly when such pain exhibits “tearing” characteristics [6].

The pathophysiological interplay between labor-induced hemodynamic stress and pre-existing vascular weaknesses is crucial in understanding how AD may be precipitated in vulnerable individuals. Increased blood pressure during contractions, alongside heightened catecholamine levels, can exacerbate underlying aortic wall weaknesses, as illustrated by a case involving a 36-year-old female with preexisting peripartum cardiomyopathy [7]. Furthermore, the literature indicates that pregnant patients with Marfan syndrome or other connective tissue disorders face significantly elevated risks for AD, emphasizing the importance of pre-conception counseling and continuous monitoring throughout pregnancy [5, 8]. Such insights suggest that labor pain management strategies, including the use of epidural analgesia, may not only enhance maternal comfort but also reduce cardiovascular risks associated with extreme hemodynamic shifts during labor [3, 9].

The diagnostic pitfalls associated with acute aortic dissection (AD) during the peripartum period are profound, particularly given the overlap of symptoms with common obstetric emergencies. This case exemplifies how severe labor pain can obscure the clinical picture of AD, resulting in potential misdiagnosis and delayed intervention. The resemblance of labor pain to more frequent obstetric complications necessitates a high index of suspicion when patients present with atypical thoracic pain, particularly when the pain exhibits characteristics such as “tearing” or radiation to the back. Previous literature has documented similar cases where initial misinterpretation of symptoms led to catastrophic outcomes, underscoring the need for vigilant evaluation in peripartum patients [9]. Furthermore, the presence of atypical pain patterns should prompt further cardiovascular investigation to rule out significant complications such as AD, especially in patients with risk factors like connective tissue disorders [10].

The pathophysiological mechanisms underlying AD during labor highlight the critical role of hemodynamic stress. The physiological changes occurring during labor, including increased blood pressure and catecholamine surges, can precipitate aortic dissection in individuals with underlying vascular vulnerabilities. This phenomenon is particularly pronounced in patients with conditions such as Marfan syndrome, where the risk of AD is significantly elevated [11]. Understanding these interactions emphasizes the importance of proactive pain management strategies, such as the use of epidural analgesia, not only for enhancing maternal comfort but also for mitigating cardiovascular risks during labor [3, 9]. Thus, integrating a multidisciplinary approach that includes obstetricians, cardiologists, and anesthesiologists is essential in optimizing outcomes for both mother and child, reiterating the need for specialized protocols in managing high-risk pregnancies [12].

The presented case underscores the critical implications of recognizing acute aortic dissection (AD) during the peripartum period, particularly reflecting on the potential misdiagnosis stemming from symptom overlap with common obstetric complications. The severe labor pain experienced by the patient was initially misattributed to typical obstetric issues, which delayed the diagnosis and intervention of a life-threatening condition. This emphasizes the necessity for heightened clinical vigilance and awareness among healthcare providers. The literature supports this perspective, revealing that atypical presentations can lead to catastrophic outcomes if not promptly addressed. Enhanced understanding of the pathophysiological mechanisms linking labor-induced hemodynamic stress and pre-existing vascular weaknesses is paramount. This case reinforces the importance of continuous cardiovascular assessment and the implementation of multidisciplinary approaches in managing such complexities, advocating for vigilant monitoring of pregnant patients, especially those with known risk factors.

Moreover, this case highlights the imperative of refining clinical protocols to improve diagnostic accuracy and patient outcomes. The integration of standardized screening measures for aortic disease in pregnant women, particularly those presenting with atypical thoracic pain, should be considered an essential practice. The collaboration among obstetricians, cardiologists, and anesthesiologists forms the backbone of effective management strategies, ensuring that both maternal and fetal well-being are prioritized during labor. Limitations of this case report include its singular nature, necessitating further studies to substantiate findings and enhance generalizability. Future research should focus on large-scale data collection to develop evidence-based guidelines for the management of AD in the peripartum setting, ultimately aiming to reduce morbidity and mortality associated with this rare but critical condition.

## Supplementary Information

Below is the link to the electronic supplementary material.

Supplementary Material 1.

## Data Availability

The original contributions presented in the study are included in the article/Supplementary material, further inquiries can be directed to the corresponding author.

## Abbreviations

AD: Aortic dissection
BP: Blood pressure
CTA: Computed tomography angiography
MDT: Multidisciplinary team
TEE: Transesophageal echocardiography
